# A novel signature based on pairwise PD‐1/PD‐L1 signaling pathway genes for predicting the overall survival in patients with hepatocellular carcinoma

**DOI:** 10.1002/ctm2.431

**Published:** 2021-05-21

**Authors:** Enfa Zhao, Shimin Chen, Ying Dang

**Affiliations:** ^1^ Department of Structural Heart Disease the First Affiliated Hospital of Xi'an Jiaotong University Xi'an China; ^2^ Department of Gastroenterology Traditional Chinese Medical Hospital of Taihe Country Taihe China; ^3^ Department of Ultrasound Medicine the First Affiliated Hospital of Xi'an Jiaotong University Xi'an China

Dear Editor,

Hepatocellular carcinoma (HCC) is ranked as the most prevalent subgroup of liver malignancies in the world, representing about 90% of primary liver cancers.^1^ To date, no widely accepted molecular biomarkers are available for survival stratification with HCC.^2^, ^3^ Recently, a novel algorithm was developed based on the relative orderings of mRNA expression patterns and had yielded excellent results.^4^, ^5^ Substantial breakthroughs have been founded in programmed death 1 (PD‐1)/ programmed death 1 ligand (PD‐L1) signaling pathway in various cancers.^6^ Here, we used the TCGA cohort to develop a signature and other two databases to confirm the prognostic model based on pairwise PD‐1/PD‐L1 signaling pathway genes.

As shown in the flowchart (Figure 1A), the mRNA expression matrixes and their clinical characteristics were retrieved from the TCGA, GEO, and the International Cancer Genome Consortium (ICGC‐JP cohort) databases. The clinicopathological information of the three databases was displayed in Table S1. Finally, 146 potential genes were acquired from the KEGG and Reactome pathway databases (Table S2). We filtered out the candidate genes computed by median absolute deviation (MAD) > 0.5. We created all possible pairs of genes based on the shared 34 genes. Among gene pairs, two genes (a and b) formed a gene pair in alphabetical order. If the expression values of gene_a_ > gene_b_ in a particular sample, the gene pair produced a score of 1; if the situation is opposite, the score equals 0. The association of these gene pairs was evaluated with univariate and LASSO Cox regression analysis after 1000 iterations (Figure 1B‐C), and finally, 12 gene pairs, included 13 genes, were identified (Table S3). The risk score was computed via the expression patterns of these pairwise genes weighted by the respective coefficient obtained from LASSO algorithm. On the basis of time‐dependent ROC curve, the ideal cutoff of the risk score was calculated at 0.573 to stratify individuals into the high‐ and low‐risk subgroups (Figure 1D). The corresponding signature risk score and individuals survival status between groups were illustrated in Figure 2A. The novel model could split individuals into low‐ and high‐risk subgroups with different outcome (Figure 2B), and with the increased risk score, individuals have a greater risk of mortality (*P* = .00197; Figure 2C). Additionally, the area under curve (AUC) of the new model for 1‐, 3‐, and 5‐year outcome in TCGA group was 0.733, 0.716, and 0.722, respectively (Figure 2D). The performance of the model was successfully verified in the two external cohorts (Figure 2E‐L). The risk score was clearly higher in individuals with increased AFP (≥300 ng/mL), advanced Tumor, Node, Metastasis (TNM) staging (stage III‐IV), advanced pathological grade (grades III‐IV), patients without family history of cancer, and patients with vascular invasion (all *P* < .05). However, there were no obviously differences in age, neoplasm status, gender, and history of prior cancer (Figure S1).

**FIGURE 1:**
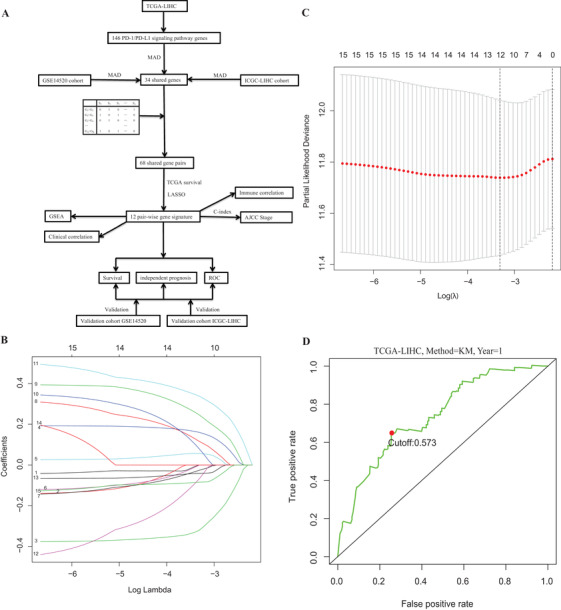
(A) A flow diagram depicting the forming and verifying of the 12‐gene pair signature. (B) LASSO coefficient profiles of the 15 survival‐related gene pairs; (C) choosing the tuning parameter (lambda) in the LASSO algorithm by 10‐fold cross‐validation; and (D) time‐dependent ROC curve illustrated the cutoff of score was determined to stratify patients into low‐ or high‐risk subgroups. Abbreviations: LASSO, least absolute shrinkage and selection operator; PD‐1, programmed death 1; ROC, area under curve; TCGA, The Cancer Genome Atlas; PD‐L1, programmed death 1 ligand

**FIGURE 2:**
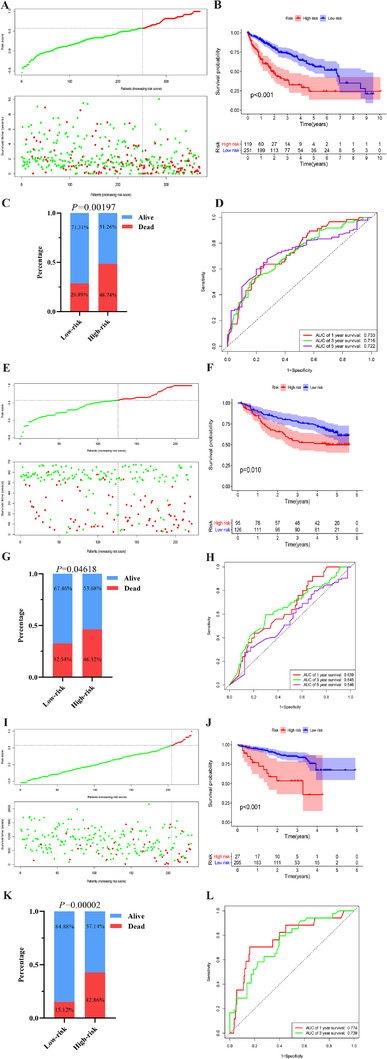
(A) The 12 pairwise genes signature corresponding risk score distribution and individuals outcome; (B) K‐M analysis curves of individuals’ OS between two groups in the TCGA cohort; (C) comparison of risk of mortality between the two groups in the TCGA cohort; and (D) time‐dependent ROC analyses of the model risk score in forecasting 1‐, 3‐, and 5‐year OS in the TCGA dataset. (E) The 12 pairwise genes signature corresponding risk score distribution and individuals outcome; (F) K‐M analysis curves of individuals’ OS between two groups in the GSE14520 dataset; (G) comparison of risk of mortality between the two groups in GSE14520 dataset; (H) time‐dependent ROC analyses of the model risk score in forecasting 1‐, 3‐, and 5‐year OS in GSE14520 dataset; (I) the 12 pairwise genes signature corresponding risk score distribution and individuals outcome in the ICGC cohort; (J) K‐M analysis curves of individuals’ OS between two groups in the ICGC portal; (K) comparison of risk of mortality between the two groups in the ICGC portal; and (L) time‐dependent ROC analyses of the model risk score in forecasting 1‐, and 3‐year survival in the ICGC portal. Abbreviations: ICGC, International Cancer Genome Consortium; K‐M, Kaplan‐Meier; OS, overall survival; ROC, area under curve; TCGA, The Cancer Genome Atlas

Multivariate Cox analysis demonstrated that the model risk score was an independent predictive indicator for OS (Figure 3A) after adjusting for multiple clinical and pathological parameters in TCGA portal. Moreover, the independent prognostic factor was verified in two validation cohorts (Figure 3B‐C). A nomogram was therefore developed (Figure 3D). The C‐index for the nomogram and TNM stage was 0.706 (95% CI: 0.648‐0.764) and 0.551 (95% CI: 0.479‐0.623), respectively. Calibration plots exhibited highly consistent with the actual observations (Figure 3E). According to decision curve analysis, the nomogram illustrated high clinical application potential and net benefits than the TNM stage examined (Figure 3F).

**FIGURE 3:**
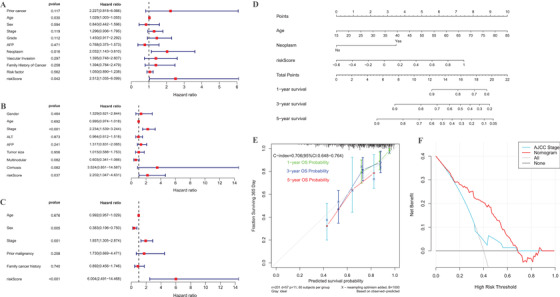
Multivariate Cox regression of the correlation between clinicopathologic factors and outcome of individuals with HCC in the TCGA portal (A), GSE14520 dataset (B), and the ICGC portal (C). Nomogram forecasting 1‐, 3‐, and 5‐year OS possibility for individuals with HCC in the TCGA portal (D); calibration curves for the nomogram (E); and DCA curves showing the comparison between the model and AJCC stage for predicting overall survival in HCC (F). Abbreviations: AJCC, American Joint Committee on Cancer; DCA, decision curve analysis; HCC, hepatocellular carcinoma; OS, overall survival; TCGA, The Cancer Genome Atlas

The outline of immune cell abundance between two subgroups measured by CIBERSORT is illustrated in Figure 4A. It was revealed that memory B cells, resting mast cells, were elevated, and CD8 T cells were reduced in high‐risk subgroup (Figure 4B‐D). The model risk score was positively associated with M0 macrophage, Tregs, neutrophil, memory B cell, eosinophil, and macrophage (all coefficient > 0 & *P* < .05) and negatively associated with monocyte, M1 macrophage, CD8+ T cell, and activated mast cell (Figure 4E). The scores of immunophenoscore (IPS), PD1/PD‐L1, CTLA4, and CTLA4 + PD1/PD‐L1 inhibitors were applied to explore the potential clinical application of immune checkpoint inhibitors. As manifested in Figure 4F‐I, the IPS, PD‐1/PD‐L1_positive scores, CTLA4_ positive, and CTLA4_ positive plus PD‐1/PD‐L1_positive scores were notably increased in the low‐risk subgroup, indicating more immunogenicity on immune checkpoint inhibitors in the low‐risk group.

**FIGURE 4:**
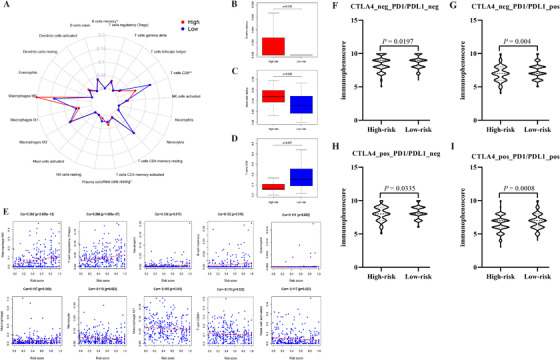
Tumor infiltrating immune cells between two subgroups and the correlation with the model risk score. Twenty two types immune cell subtypes between two subgroups (A); the abundance distribution of memory B cells (B), resting mast cells (C), and CD8 T cells between high‐ and low‐risk subgroups; (D) correlation determined by Person correlation analysis between the signature risk score and infiltrating immune cells in patients with HCC (*P* < .05). IPS comparison between two subgroups in patients with HCC in CTLA4_neg_PD1/PDL1_neg (E), CTLA4_negative + PD‐1/PD‐L1_positive (F), CTLA4_ positive + PD‐1/PD‐L1_ negative (G), and CTLA4_ positive + PD‐1/PD‐L1_positive (H); PD‐1/PD‐L1_pos or CTLA4_pos indicated anti‐ PD‐1/PD‐L1 or anti‐CTLA4 therapy, respectively. Abbreviations: CTLA4, cytotoxic T‐lymphocyte associated protein 4; IPS, immunophenoscore; PD‐1, programmed death 1; PD‐L1, programmed death 1 ligand

Gene ontology (GO) analysis of the 13 signaling pathway genes illustrated that the signature was mainly enriched in immune response processes (Figure S2A). KEGG enrichment analysis demonstrated that the signature was mainly involved in PD−L1/PD−1 checkpoint pathway in cancer, Fc epsilon RI signaling pathway, and B‐/T‐cell receptor signaling pathways (Figure S2B). GSEA performed between two risk subgroups exhibited that multiple gene sets were markedly involved in high‐risk group (Figure S2C) and several metabolism pathways were mainly involved in low‐risk group (Figure S2D).

Traditional approaches that previous studies used were required scaling and appropriate normalization among various measurement platforms.^2^, ^7^ We designed a novel method for the identification of functional associations between pairwise genes based on relative expression orderings of two genes within‐sample, without the need of standardization, batch correction and scaling among different platforms. Besides, the cutoff of the risk score can be applied to other validation cohort in the future because it was computed as expression levels (0 or 1) of these pairwise genes weighted by the coefficients, presenting some significant advantages than previous signatures. Moreover, to identify reliable signature of HCC prognosis, we developed a signature for patients with HCC and confirmed its performance in other two external validation cohorts with large cancer samples for biomarker mining through multidimensional bioinformatics methods. The signature was accurate and robust, giving that the data resource was adequate and the analytical procedures were comprehensive. Therefore, all findings reveal that the new model can present a precise overall survival (OS) prediction of HCC patients and can measure HCC OS in an individualized way and can be readily translated into clinical utility according to the cutoff value determined from previous formula.

In conclusion, we proposed and validated a pairwise PD‐1/PD‐L1 gene panel that can accurately predict OS of patients with HCC. The signature can help to the effect of immunotherapy for HCC patients and be used as a promising marker to evaluate individuals who could benefit from immunotherapy.

## ETHICS APPROVAL AND CONSENT TO PARTICIPATE

The data used were publicly available to all, so the ethics approval was waived.

## DATA AVAILABILITY STATEMENT

All datasets that support the present study are publicly available to all at GEO (https://www.ncbi.nlm.nih.gov/geo/) database, ICGC portal, and the TCGA portal.

## CONFLICT OF INTEREST

None

## Supporting information


**Figure S1** The relationships between model risk score and multiple clinicopathologic features in the TCGA portal

**Figure S2** Functional enrichment analysis using 13 included signaling pathway genes; (A) GO analysis and (B) KEGG pathways gene functional enrichment of 13 unique genes; GASE demonstrated the remarkably enriched KEGG pathways in TCGA in the high‐risk subgroup (C) and low‐risk subgroup (D)

Supporting Information

Supporting Information

Supporting Information
