# Supplementary figures and images for: A novel signature based on pairwise PD‐1/PD‐L1 signaling pathway genes for predicting the overall survival in patients with hepatocellular carcinoma
Source: Clin Transl Med. 2021 May 21;11(5):e431. doi: 10.1002/ctm2.431 (PMC8140183; doi:10.1002/ctm2.431)

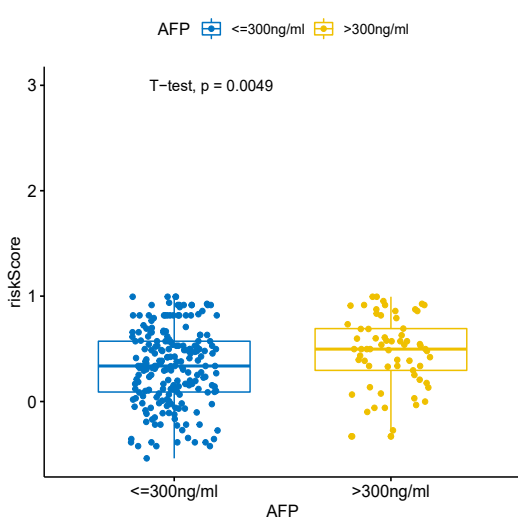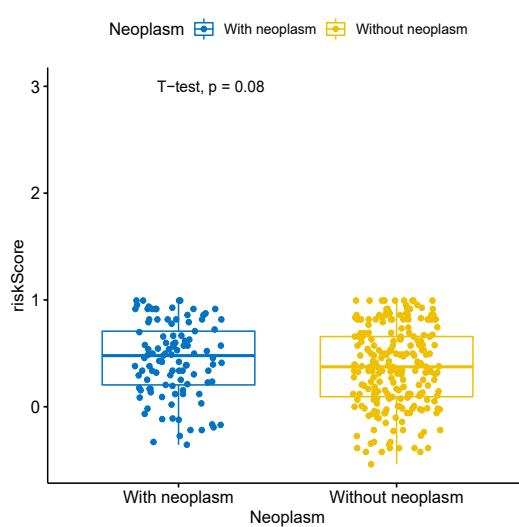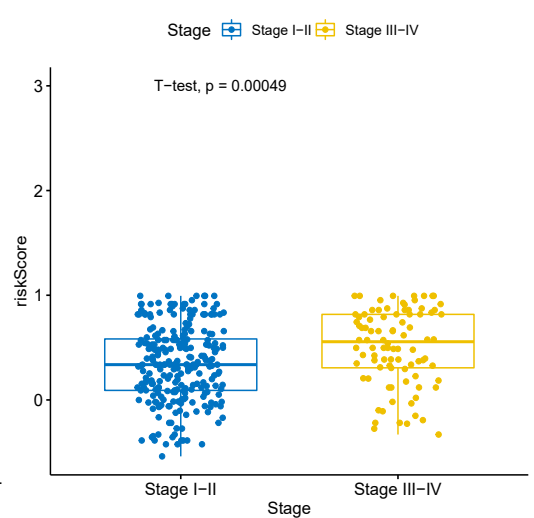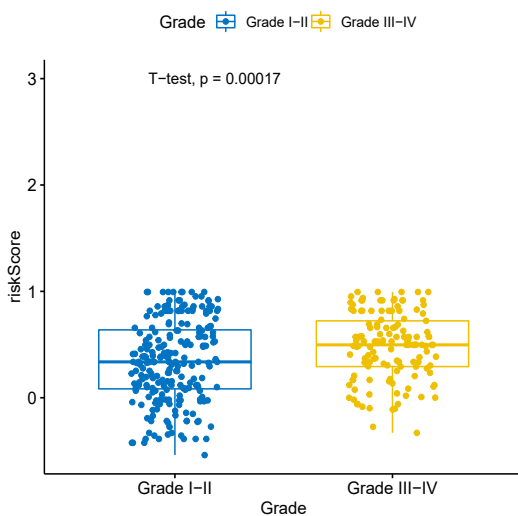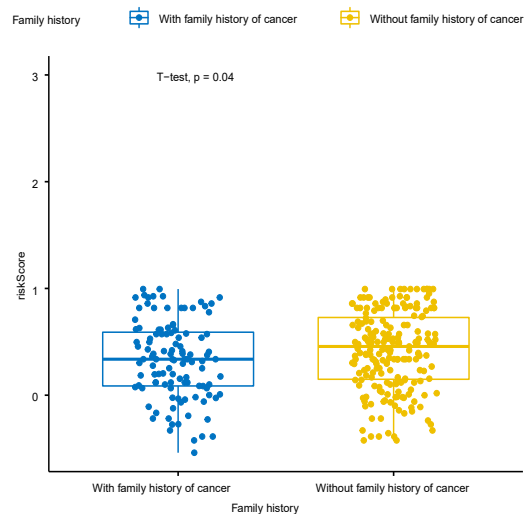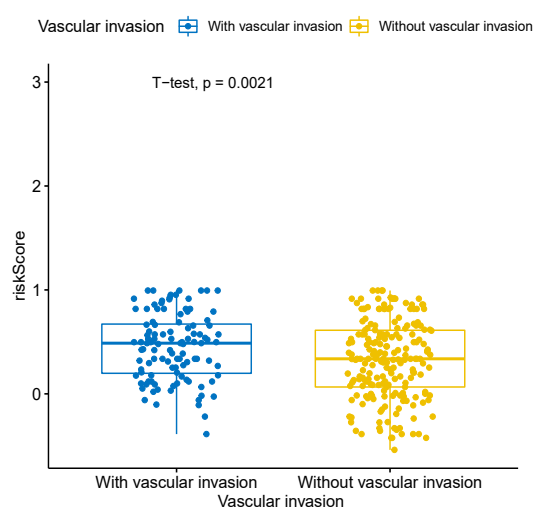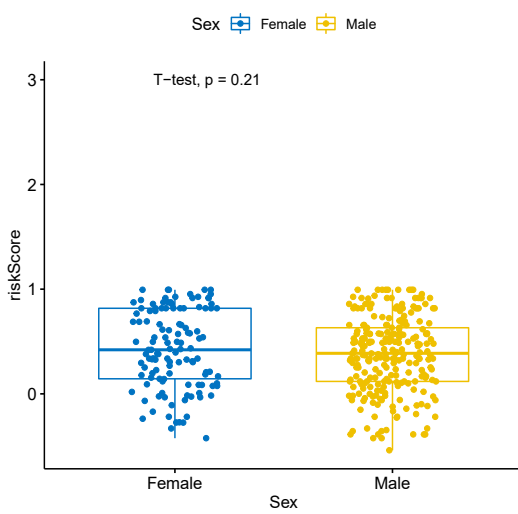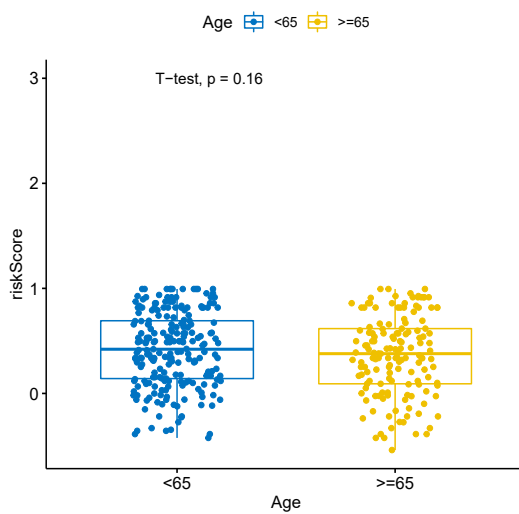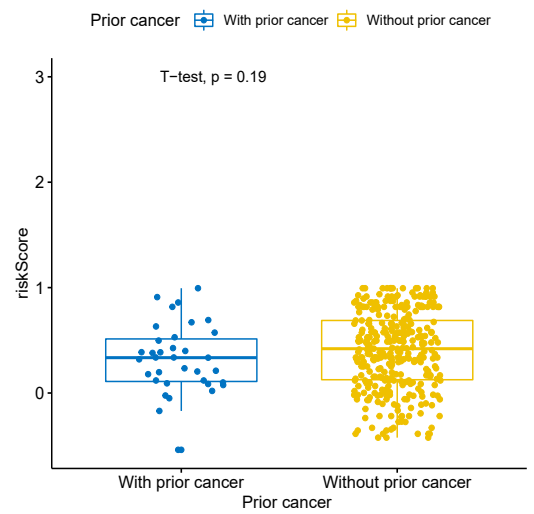

Supplement: Supplementary file 1 — Figure S1 The relationships between model risk score and multiple clinicopathologic features in the TCGA portal [file CTM2-11-e431-s005.pdf]
