# Supplementary material for: A novel signature based on pairwise PD‐1/PD‐L1 signaling pathway genes for predicting the overall survival in patients with hepatocellular carcinoma
Source: Clin Transl Med. 2021 May 21;11(5):e431. doi: 10.1002/ctm2.431 (PMC8140183; doi:10.1002/ctm2.431)

A

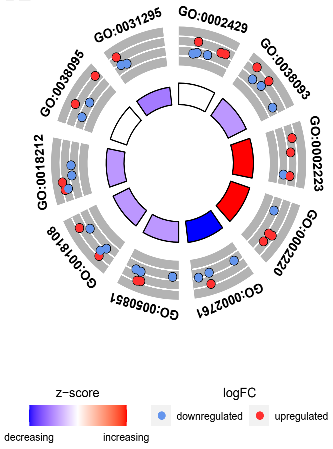

ID	Description
GO:0002429	immune response−activating cell surface receptor signaling pathway
GO:0038093	Fc receptor signaling pathway
GO:0002223	stimulatory C−type lectin receptor signaling pathway
GO:0002220	innate immune response activating cell surface receptor signaling pathway
GO:0002761	regulation of myeloid leukocyte differentiation
GO:0050851	antigen receptor−mediated signaling pathway
GO:0018108	peptidyl−tyrosine phosphorylation
GO:0018212	peptidyl−tyrosine modification
GO:0038095	Fc−epsilon receptor signaling pathway
GO:0031295	T cell costimulation

B

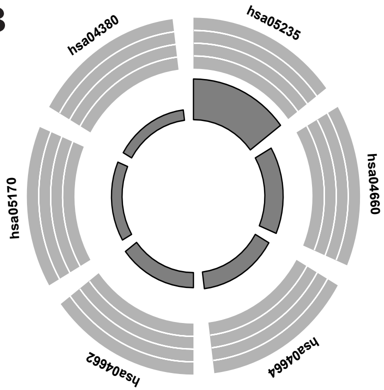

ID	Description
hsa05235	PD−L1 expression and PD−1 checkpoint pathway in cancer
hsa04660	T cell receptor signaling pathway
hsa04664	Fc epsilon RI signaling pathway
hsa04662	B cell receptor signaling pathway
hsa05170	Human immunodeficiency virus 1 infection
hsa04380	Osteoclast differentiation

C

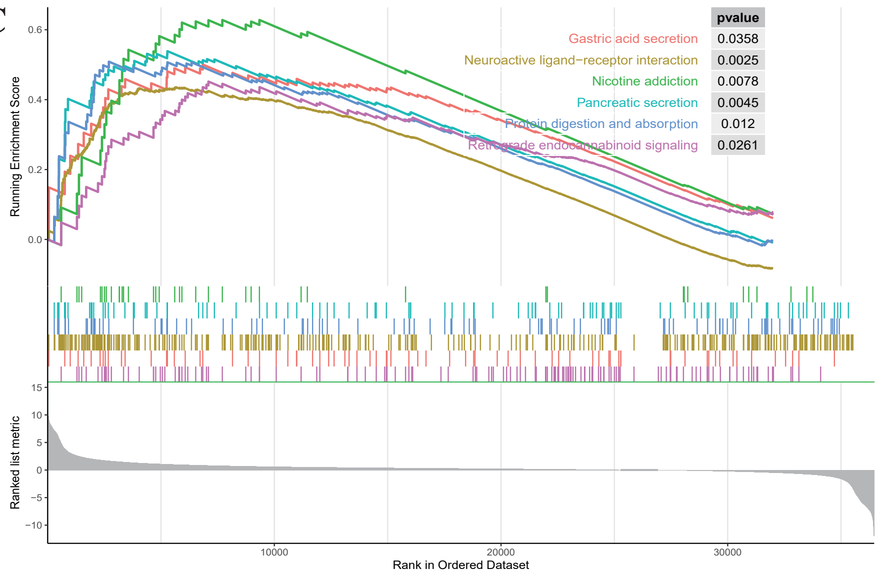

D

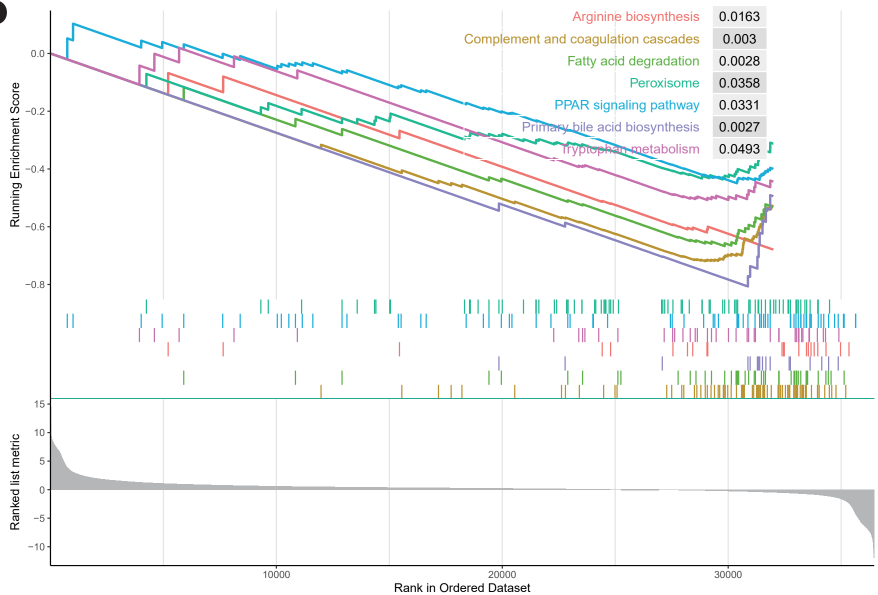

Supplement: Supplementary file 2 — Figure S2 Functional enrichment analysis using 13 included signaling pathway genes; (A) GO analysis and (B) KEGG pathways gene functional enrichment of 13 unique genes; GASE demonstrated the remarkably enriched KEGG pathways in TCGA in the high‐risk subgroup (C) and low‐risk subgroup (D) [file CTM2-11-e431-s002.pdf]
