# Supplementary material for: A novel signature based on pairwise PD‐1/PD‐L1 signaling pathway genes for predicting the overall survival in patients with hepatocellular carcinoma
Source: Clin Transl Med. 2021 May 21;11(5):e431. doi: 10.1002/ctm2.431 (PMC8140183; doi:10.1002/ctm2.431)
Supplement: Supplementary file 3 — Supporting Information [file CTM2-11-e431-s001.docx]

**Table S1** The clinicopathological characteristics of the three cohorts

| Variables | Subgroups | TCGA(N=370) | GSE14520(N=221) | ICGC(N=232) |
| --- | --- | --- | --- | --- |
| Age |  |  |  |  |
|  | <60 | 169 | 178 | 45 |
|  | >=60 | 201 | 43 | 187 |
| Sex |  |  |  |  |
|  | Male | 249 | 191 | 171 |
|  | Female | 121 | 30 | 61 |
| Stage |  |  |  |  |
|  | I | 171 | 93 | 36 |
|  | II | 85 | 77 | 106 |
|  | III | 85 | 49 | 71 |
|  | IV | 5 | 2 | 19 |
|  | Unknown | 24 | 0 | 0 |
| Grade |  |  |  |  |
|  | I | 55 | - | - |
|  | II | 177 | - | - |
|  | III | 121 | - | - |
|  | IV | 12 | - | - |
|  | NA | 5 | - | - |
| Survival status |  |  |  |  |
|  | Dead | 130 | 85 | 43 |
|  | Living | 240 | 136 | 189 |
| Vascular invasion |  |  |  |  |
|  | Positive | 108 | - | - |
|  | Negative | 206 | - | - |
|  | Unknown | 56 | - | - |
| Family history |  |  |  |  |
|  | Positive | 112 | - | 74 |
|  | Negative | 207 | - | 143 |
|  | Unknown | 51 | - | 15 |
| Prior malignancy |  |  |  |  |
|  | Positive | 35 | - | 30 |
|  | Negative | 335 | - | 202 |
|  | Unknown | 0 | - | 0 |
| AFP |  |  |  |  |
|  | < 300 ng/mL | 212 | 118 | - |
|  | ≥300 ng/mL | 65 | 100 | - |
|  | Unknown | 93 | 3 | - |
| HCC risk factors |  |  |  |  |
|  | No history of risk factors | 91 | - | - |
|  | Alcohol consumption | 68 | - | - |
|  | Hepatitis B | 75 | 221 | - |
|  | Hepatitis C | 32 | - | - |
|  | Alcohol consumption/Hepatitis B/Hepatitis C | 38 | - | - |
|  | Other | 47 | - | - |
|  | Unknown | 19 | - | - |
| Tumor size |  |  |  |  |
|  | ≤5cm | - | 140 | - |
|  | >5cm | - | 80 | - |
|  | Unknown | - | 1 | - |
| ALT |  |  |  |  |
|  | >50 U/L | - | 91 | - |
|  | ≤50 U/L | - | 130 | - |
| Multinodular |  |  |  |  |
|  | Yes | - | 45 | - |
|  | No | - | 178 | - |
| Cirrhosis |  |  |  |  |
|  | Yes | - | 18 | - |
|  | No | - | 203 | - |
