# Supplementary material for: A novel signature based on pairwise PD‐1/PD‐L1 signaling pathway genes for predicting the overall survival in patients with hepatocellular carcinoma
Source: Clin Transl Med. 2021 May 21;11(5):e431. doi: 10.1002/ctm2.431 (PMC8140183; doi:10.1002/ctm2.431)
Supplement: Supplementary file 4 — Supporting Information [file CTM2-11-e431-s003.docx]

**Table S2** 146 PD-1/PD-L1 signaling pathway genes

| TNFRSF14 | MLST8 | HLA-DRB1 | PPP2R5E | NFKB1 | CSNK2B |
| --- | --- | --- | --- | --- | --- |
| BTLA | TRIB3 | HLA-DQB1 | PPP2CA | RELA | ZAP70 |
| PTPN6 | THEM4 | HLA-DRB3 | PPP2CB | IFNG | MAP3K3 |
| PTPN11 | MAP3K8 | HLA-DQB2 | HIF1A | IFNGR1 | MAP2K3 |
| ICOS | MAP3K14 | HLA-DPB1 | EGF | IFNGR2 | MAP2K6 |
| ICOSLG | SRC | HLA-DRB5 | EGFR | JAK1 | MAPK11 |
| CD80 | YES1 | HLA-DRB4 | HRAS | JAK2 | MAPK12 |
| CD86 | LYN | CD3E | KRAS | STAT1 | MAPK13 |
| CD28 | GRB2 | CD3D | NRAS | STAT3 | MAPK14 |
| FYN | VAV1 | TRBV12-3 | RAF1 | TLR2 | LAT |
| LCK | RAC1 | TRBV7-9 | MAP2K1 | TLR4 | PLCG1 |
| GRAP2 | CDC42 | TRBC1 | MAP2K2 | TLR9 | PPP3CA |
| AKT2 | PAK3 | TRAV8-4 | MAPK1 | TIRAP | PPP3CB |
| AKT3 | PAK2 | TRAV29DV5 | MAPK3 | MYD88 | PPP3CC |
| AKT1 | PAK1 | TRAV19 | FOS | TRAF6 | PPP3R1 |
| PDPK1 | CSK | TRAC | JUN | NFATC1 | PPP3R2 |
| PIK3CA | CD274 | CD3G | EML4 | NFATC2 | RASGRP1 |
| PIK3R3 | PDCD1LG2 | CD247 | ALK | NFATC3 | PRKCQ |
| PIK3R2 | PDCD1 | CTLA4 | PIK3CD | TICAM1 | IKBKG |
| PIK3R1 | CD4 | PPP2R1A | PIK3CB | TICAM2 | NFKBIA |
| MAPKAP1 | HLA-DPA1 | PPP2R1B | PTEN | BATF3 | NFKBIB |
| RICTOR | HLA-DQA1 | PPP2R5B | RPS6KB1 | BATF |  |
| MTOR | HLA-DRA | PPP2R5C | RPS6KB2 | BATF2 |  |
| PRR5 | HLA-DQA2 | PPP2R5A | CHUK | CSNK2A1 |  |
| PPP2R5D | IKBKB | CSNK2A2 | NFKBIE | CSNK2A3 |  |
