# Supplementary material for: A novel signature based on pairwise PD‐1/PD‐L1 signaling pathway genes for predicting the overall survival in patients with hepatocellular carcinoma
Source: Clin Transl Med. 2021 May 21;11(5):e431. doi: 10.1002/ctm2.431 (PMC8140183; doi:10.1002/ctm2.431)
Supplement: Supplementary file 5 — Supporting Information [file CTM2-11-e431-s004.docx]

**Table S3.** The detailed information of the 12 pair-wise PD-1/PD-L1 signaling pathway genes and the coefficient obtained from the least absolute shrinkage and selection operator (LASSO) regression analysis

| Gene pair 1 | Full name | Gene pair 2 | Full name | Coefficient |
| --- | --- | --- | --- | --- |
| CD4 | CD4 molecule | NRAS | NRAS proto-oncogene, GTPase | -0.320136109 |
| CHUK | component of inhibitor of nuclear factor kappa B kinase complex | FYN | FYN proto-oncogene, Src family tyrosine kinase | 0.160101321 |
| CSNK2A1 | casein kinase 2 alpha 1 | MAP2K3 | mitogen-activated protein kinase kinase 3 | 0.056806147 |
| FOS | Fos proto-oncogene, AP-1 transcription factor subunit | NRAS | NRAS proto-oncogene, GTPase | -1.01E-05 |
| FYN | FYN proto-oncogene, Src family tyrosine kinase | LYN | LYN proto-oncogene, Src family tyrosine kinase | -0.025722029 |
| IFNGR2 | interferon gamma receptor 2 | PIK3R1 | phosphoinositide-3-kinase regulatory subunit 1 | 0.115524921 |
| IFNGR2 | interferon gamma receptor 2 | PPP2R1B | protein phosphatase 2 scaffold subunit Abeta | 0.245384239 |
| MAP2K1 | mitogen-activated protein kinase kinase 1 | MAP2K3 | mitogen-activated protein kinase kinase 3 | 0.078629825 |
| MAP2K1 | mitogen-activated protein kinase kinase 1 | PIK3R1 | phosphoinositide-3-kinase regulatory subunit 1 | 0.337653105 |
| MAP2K1 | mitogen-activated protein kinase kinase 1 | TRIB3 | tribbles pseudokinase 3 | -0.067339801 |
| MAP2K3 | mitogen-activated protein kinase kinase 3 | NRAS | NRAS proto-oncogene, GTPase | -0.04133876 |
| PPP2R1B | protein phosphatase 2 scaffold subunit Abeta | TRIB3 | tribbles pseudokinase 3 | -0.084388039 |
